# Investigation of fructose consumption on hippocampal insulin and glucagon-like peptide-1 receptors, and metabolic effects in rats

**DOI:** 10.22038/IJBMS.2023.70711.15369

**Published:** 2023

**Authors:** Fatih Altintas, Sadettin Caliskan, Melek Tunc-Ata, Emine Kilic-Toprak, Onur Tokgun, Neslihan Esra Avci, Vural Kucukatay

**Affiliations:** 1 Department of Physiology, Faculty of Medicine, Pamukkale University, Denizli, Turkey; 2 Department of Physiology, Faculty of Medicine, Üsküdar University, İstanbul, Turkey; 3 Department of Medical Genetics, Faculty of Medicine, Pamukkale University, Denizli, Turkey; 4 Department of Physiology, Faculty of Medicine, İzmir Demokrasi University, İzmir, Turkey

**Keywords:** Fructose, Glucagon-like peptide-1 Hippocampus, Insulin, Lipid profile

## Abstract

**Objective(s):**

The detrimental effects of high fructose consumption on metabolic health have been extensively studied. However, limited research has focused on the impact of fructose intake on neuroprotective mechanisms, specifically the expression of insulin receptor (INSR) and glucagon-like peptide-1 receptor (GLP-1R) in the hippocampus. Understanding the effects of fructose on these neuroprotective molecules can provide valuable insights into the potential role of fructose in hippocampal dysfunction. The goal of this study is to aim at the basal plasma levels of lipid profile, insulin, GLP-1, and HOMA-IR, as well as the mRNA and protein expression of neuroprotective molecules such as INSR and GLP-1R in Wistar rats fed a high fructose diet.

**Materials and Methods:**

Rats were separated into control (C) and high fructose (HF) groups. The HF group was given 20% fructose water to drink for 16 weeks.

**Results:**

Fructose ingestion significantly increased abdominal fat (C=1.24±0.08 g, HF=1.79±0.19 g, *P<*0.05) and plasma triglyceride levels (C=179.22±22.85 µg/ml, HF=242.45±14.45 µg/ml, *P<*0.05), but had no statistically significant effect on body weight and plasma HDL, LDL, total cholesterol, insulin, and GLP-1 levels (*P>*0.05). Although INSR mRNA expression in the hippocampus was significantly lower in the HF group compared to the control group (*P<*0.05), GLP-1R mRNA expression did not differ significantly across the groups (*P>*0.05). Furthermore, whereas INSR and GLP-1R protein levels in the experimental group were on a declining trend, this trend was not substantially different (*P>*0.05).

**Conclusion:**

These data suggest that fructose consumption may be harmful to the hippocampus by lowering the expression of INSR.

## Introduction

Fructose is a naturally occurring monosaccharide found in fruits and vegetables. It is also present in processed foods and drinks (1). Since the 1970s, fructose consumption has been steadily increasing (2). Daily consumption in the United States has climbed from 16-20 g to 60-150 g in recent years (3). Consumption of a high fructose diet has been proven to produce substantial metabolic alterations both systemically and in particular organs (4, 5). Currently, fructose and its metabolites such as lactate, trioses-phosphate, etc. have been discovered as harmful compounds that may contribute to the development of illnesses such as obesity, dyslipidemia, insulin resistance, type 2 diabetes, cardiovascular disease, and chronic kidney disease (6, 7). Indeed, epidemiological studies have demonstrated an association between excessive fructose consumption and insulin resistance, obesity, dyslipidemia, metabolic syndrome, type 2 diabetes mellitus, and cardiovascular disease in people and animals (8-10). The mechanism by which a high fructose diet adversely affects several organs and tissues is not entirely known (11). 

The brain is one of the organs most damaged by fructose poisoning. Fructose and its metabolites have been shown to influence the brain. For instance, it is suggested that even a one-week period of fructose ingestion might alter brain plasticity (12). A high fructose diet has been shown to dramatically impair neural progenitor cell proliferation, differentiation, and survival of newborn neurons (13). The number of contact sites in the hippocampus, the size of postsynaptic densities, and hippocampal neurogenesis were all decreased in mice fed fructose (14). Although much research has been published in this area, the mechanism by which fructose consumption causes these adverse consequences has not been thoroughly explored. 

The glucagon-like peptide-1 receptor (GLP-1R) has been revealed to be expressed in different brain areas, including the hypothalamus, hippocampus, and neocortex (15, 16), and glucagon-like peptide-1 (GLP-1) has been demonstrated to have neuroprotective effects in the brain (17). Indeed, research on GLP-1R mutant mice found that learning, memory, and synaptic plasticity are reduced (18). These findings suggest that GLP-1 may play a critical role in neuroprotection. Along with GLP-1, insulin receptor (INSR) signaling is crucial for brain development, neuroprotection, metabolism, and plasticity (19). It has been demonstrated that INSR deficits can result in a wide variety of brain problems, including neurodevelopmental syndromes and neurodegenerative diseases (19). Additionally, GLP-1 has been shown to have a function in the control of the INSR on neuronal membranes. For example, it has been demonstrated that activating the GLP-1R with liraglutide, a GLP-1R agonist, partially normalizes the lowered INSR level and altered synaptic structure observed in Alzheimer’s disease (20). Additionally, GLP-1 has been shown to alleviate brain insulin resistance (21). Taken together, alterations in one or more of the aforementioned variables may represent a plausible mechanism of fructose-mediated neurotoxicity.

The goal of this study is to see how the above-mentioned molecules, which play important roles in neuroprotection, change in hippocampus tissue after excessive fructose consumption. For this purpose, we wanted to look at INSR and GLP-1R mRNA levels as well as INSR and GLP-1R protein levels in the hippocampus. We also wanted to see how increased fructose consumption affected plasma basal insulin and GLP-1 levels, as well as glucose, insulin resistance, lipid profiles, and abdominal obesity.

## Materials and Methods

***Animals and experimental protocol***

The Pamukkale University Experimental Surgery Application and Research Center provided 2-3 month old Wistar rats. All animal handling and experimental techniques followed the National Institutes of Health Guidelines for the Care and Use of Laboratory Animals and were approved by Pamukkale University’s Animal Care and Use Ethics Committee (06/07/2018/60758568-020/46214). Rats were kept in groups of four to five per cage (42×26×15 cm) in a room with a regulated temperature (23±2 ^°^C**) **and relative humidity (60±5%) with lights on from 7:00 to 19:00. The rats were divided into two groups: control (C, n=9) and high fructose (HF, n=10). While rats in the HF group received 20% fructose in their drinking water (20 g fructose in 100 ml tap water) for 16 weeks, rats in the C group received tap water. All rodents were fed regular rat *chow ad libitum*. 

At the end of the sixteenth week, the animals were fasted for 12 hr, and blood was drawn from the rats’ tail veins for fasting blood sugar measurements. Heparinized blood was collected from the abdominal aorta of rats under anesthesia with ketamine and xylazine (90 mg/kg; 10 mg/kg, respectively). Plasma was collected from cells by centrifugation at 3000 rpm for 10 min, which was utilized to assess insulin, GLP-1, and lipid profiles. At the end of the study, rats were killed by exsanguination. Surgically, the adipose tissue covering the testis (perigonadal fat) and the adipose tissue on the muscle behind the abdominal cavity (retroperitoneal fat) were collected. All collected fat was termed “abdominal fat” and the amounts of fat per 100 g of body weight were calculated. 

The right hippocampus was removed immediately from the rats and homogenized in Trizol solution for PCR analysis. The left hippocampus was homogenized in a radioimmunoprecipitation assay (RIPA) solution for protein quantification. Homogenates were held at -20 and -80 degrees Celsius, respectively. 

***Plasma biochemical parameters***

The levels of fasting triglyceride, total cholesterol, high-density lipoprotein (HDL), and low-density lipoprotein (LDL) were determined by commercial kits (Cloud-Clone for triglyceride, BTLab for others). Furthermore, total plasma levels of GLP-1 were measured (Cloud-Clone). Glucose was measured using a handheld glucometer (ACCU-CHEK Performa Nano). Plasma insulin was measured using a rat insulin ELISA kit (Elabscience). Lee index was calculated ((body weight (g)^^1/3^/naso-anal length (cm))×1000) to evaluate the growth performance of the rats and the development of obesity. The HOMA-IR index was calculated according to the following equation: HOMA-IR= fasting plasma glucose (mmol/l)×fasting plasma insulin (mIU/l)/22.5.

***Total RNA isolation, cDNA synthesis, and real-time polymerase chain reaction***

Total RNA was obtained with Trizol reagent (Invitrogen) from the hippocampus tissue. Then, cDNA was synthesized with the commercial High-Capacity cDNA Reverse Transcription Kit (Applied Biosystems). INSR and GLP-1R gene expressions were analyzed by real-time PCR method using PowerUp SYBR Green Master Mix (Applied Biosystems). The relative change of gene expressions was analyzed according to the β-actin reference/housekeeping gene. Primer sequences used in real-time PCR were shown in Table 1. The real-time PCR protocol was applied as follows: 2 min at 50 ^°^C, 2 min at 95 ^°^C for one cycle and 15 sec at 95 ^°^C, 15 sec at 60 ^°^C, and 1 min at 72 ^°^C for 40 cycles. Relative changes in target genes were analyzed with the help of the reference gene. Then, the calculation was made with the formula 2^^-∆∆Ct^. According to the results, relative gene expressions between groups were found.

***Protein expressions with Western-blot method***

Hippocampus tissue was homogenized in RIPA buffer with the presence of a protein inhibitor cocktail (Santa Cruz Biotechnology, sc-24948) on ice freshly. After centrifugation at 10,000 rpm and 4 ^°^C, a BioRad DC Protein assay kit (Bradford dye-binding procedure) was used to calculate the protein concentration by using supernatant. The isolated protein samples (50 µg each) were subjected to sodium dodecyl sulfate-polyacrylamide gel (SDS-PAGE) and then passed to the PVDF membrane (Millipore) with a semi-dry transfer system (BioRad). The PVDF membrane containing the proteins was incubated in freshly prepared PBS+5% skim milk powder+0.05% Tween-20 for 1 hr at room temperature. After PBS with 0.05% Tween-20 (PBST) washes, INSR (rabbit polyclonal 1:1000, Elabscience), GLP-1R (rabbit polyclonal 1:1000, Bioss) or β-actin (rabbit polyclonal 1:1000, BTLab) primary antibodies were incubated at room temperature for 2 hr. After PBST washes, horseradish peroxidase (HRP)-conjugated secondary antibody (goat anti-rabbit IgG 1:10000, Elabscience) was incubated for 30 min. The results were visualized by the enhanced chemiluminescence detection technique. The bands were analyzed with the ImageJ program. β-actin was used as an internal reference protein.

***Statistical method***

The data were analyzed with SPSS 25.0 (IBM Corp., Armonk, NY, USA) package program. Continuous variables are expressed as mean±standard error (SE). The suitability of the data to normal distribution was examined by Shapiro-Wilk tests. When parametric test assumptions are provided, independent samples t-test was used to compare independent group differences. When parametric test assumptions are not provided, the Mann-Whitney U test was used to compare independent group differences. In all analyses, *P*<0.05 was considered statistically significant. 

## Results

***Lee index, abdominal obesity***

**Figure 1:**
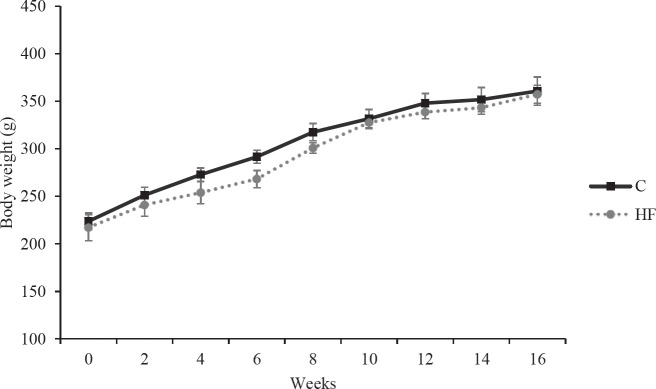
Weekly change of body weight of Wistar rats

**Figure 2:**
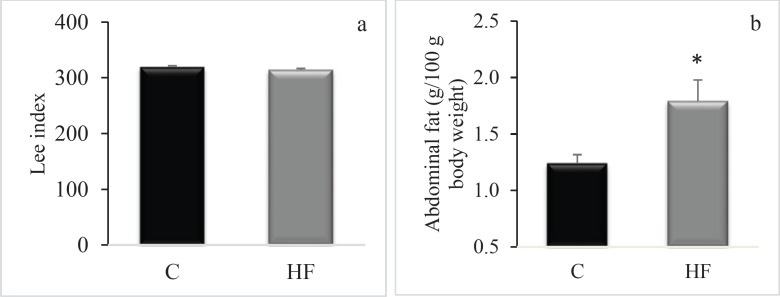
Effect of the fructose consumption on (a) Lee index and (b) abdominal fat amounts (per 100 g body weight) in Wistar rats

Along with the experiment, there was no significant difference in the body weights of rats in both groups (Figure 1). The body weights of the rats at the start and end of the study were as follows: C=223.7±8.7 g, HF=217.0±13.8 g; C=360.7±14.9 g, HF=357.3±9.5 g, respectively, *P*>0.05 for both. While there was no change in lee index between the HF and control groups (C=319±2.5, HF=314±2.4, *P*>0.05, Figure 2a), there was a statistically significant increase in abdominal fat mass (retroperitoneal and perigonadal) in the HF group (C=1.24±0.08 g, HF=1.79±0.19 g, *P*<0.05, Figure 2b).

***Fasting glucose, insulin, HOMA-IR score, and lipid profile***

The high fructose consumption did not result in a significant change in the HOMA-IR score, plasma fasting insulin, GLP-1, or glucose levels in the experimental groups as compared to the control group (*P*>0.05). Additionally, there was no difference in HDL, LDL, or total cholesterol levels between the groups (*P*>0.05). Only the triglyceride level was shown to be significantly higher in the HF group compared to the control group (*P*<0.05). All plasma biochemical results were shown in Table 2.

**Figure 3:**
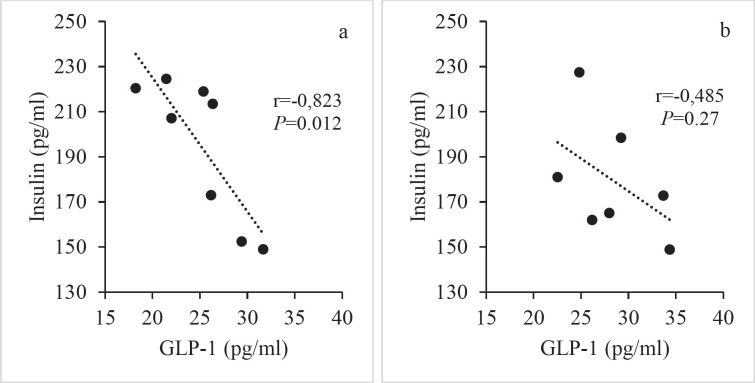
Pearson’s correlation analysis of fasting plasma GLP-1 and insulin in (a) C group and (b) HF group in Wistar rats

Pearson’s correlation analysis indicated that there was a strong, negative, and significant correlation between insulin and GLP-1 in the C group (r=-0.823, n=8, *P*=0.012) (Figure 3a). This negative and significant correlation between insulin and GLP-1 was not observed in the HF group (r=-0.485, n=7, *P*=0.27) (Figure 3b).

***Hippocampus INSR and GLP-1R mRNA and protein expressions***

**Figure 4:**
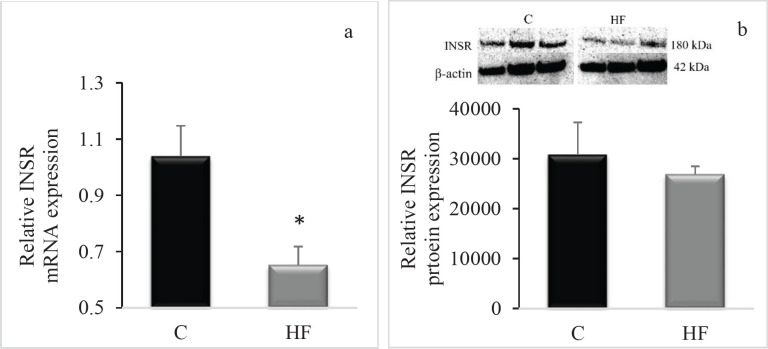
Effect of the fructose consumption on (a) hippocampal INSR mRNA expression, (b) hippocampal INSR protein expression in Wistar rats

**Figure 5:**
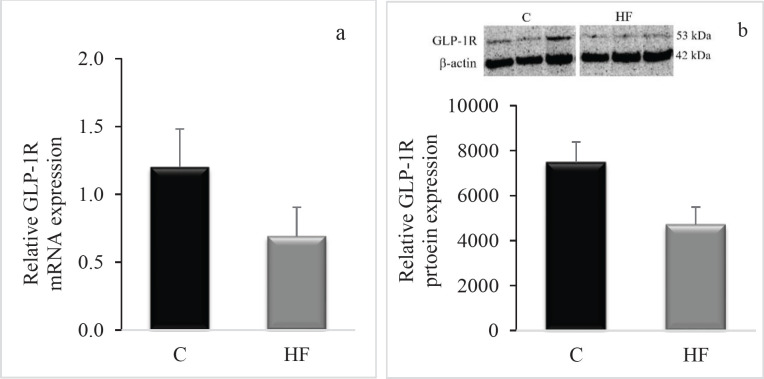
Effect of fructose consumption on (a) hippocampal GLP-1R mRNA expression and (b) hippocampal GLP-1R protein expression in Wistar rats

When the HF group was compared to the control group, there was a statistically significant difference in hippocampal INSR mRNA levels (*P*=0.001, Figure 4a). Similar to the INSR mRNA, a tendency toward a decrease in INSR protein level was detected; however, this trend was not statistically significant (*P*>0.05, Figure 4b). Unlike the INSR, despite a trend for lower hippocampal GLP-1R mRNA and protein levels in the HF group compared to the control group (Figure 5a, 5b), these decreases were not statistically significant (*P*>0.05).

**Table 1:** List of primer sequences used for gene expression analysis by real-time PCR

Primer name	Forward	Reverse	Product length
**β** **-actin**	5′-CGTTGACATCCGTAAAGACC-3′	5′-GCCACCAATCCACACAGA-3′	172
**INSR**	5’-CAGTGTCGTGATCGGAAGTATT-3’	5’-CTGAGGTACTCTGGGTTTGAAG-3′	99
**GLP-1R**	5′-CCGCTTCTGGGCACGCATGA-3′	5′-AGCGCTCCCAGCTCTTCCGA-3′	187

β-actin: beta-actin; INSR: insulin receptor; GLP-1R: glucagon-like peptide-1 receptor

Data are the mean±standard error. Data analysis was carried out by independent samples t-test

C: control; HF: high fructose (n=9-10)

**Table 2:** Plasma biochemical levels of Wistar rats in control (C) and high fructose (HF) groups

	**Control (C)**	**High fructose (HF)**	***P***
Triglyceride (µg/ml)	179,22 ± 22,85	242,45 ± 14,45	0,03*
HDL (mmol/l)	1,99 ± 0,12	2,07 ± 0,12	0,62
LDL (ng/ml)	95,89 ± 5,48	91,17 ± 4,85	0,53
Total cholesterol (mmol/l)	7,03 ± 0,23	7,46 ± 0,20	0,18
Insulin (pg/ml)	194,89 ± 11,17	179,39 ± 9,95	0,33
Glucose (mg/dl)	106,78 ± 3,54	106,1 ± 6,04	0,93
HOMA-IR	1,47 ± 0,09	1,43 ± 0,16	0,83
GLP-1 (pg/ml)	24,84 ± 1,39	28,24 ± 1,58	0,13

Data are the mean±standard error. *: significance between groups

Data are the mean±standard error. *P<*0.05, data analysis was carried out by independent samples t-test. *: significance between groups

C: control; HF: high fructose (n=9-10)

C: control; HF: high fructose (n=7-8)

Data are the mean ± standard error. *P<*0.05, data analysis was carried out mann whitney u test. *: significance between groups

C: control; HF: high fructose; INSR: insulin receptor (n=4-10)

Data are the mean±standard error. *P<*0.05, data analysis was carried out with independent samples t-test for “a” and Mann-Whitney u-test for “b”

C: control; HF: high fructose; GLP-1R: glucagon-like peptide-1 receptor (n=4-9)

## Discussion

The current work demonstrates that fructose ingestion has a notable influence on the levels of neuroprotective molecules in the rat hippocampus, including INSR. We demonstrated that fructose ingestion dramatically decreased hippocampus INSR mRNA expression. This is the first study that we are known of that examines the impact of a 16-week-high-fructose diet on INSR expression in a rat model. According to our results, when compared to the control group, high fructose consumption was shown to increase the amount of abdominal fat statistically significantly in rats, but it had no influence on body weight or the Lee Index, which is an indication of obesity in rodents. Similarly, although no significant change was seen in body weight in rats given fructose water (22), it was reported that 20% fructose ingestion resulted in increased calorie intake and abdominal fat tissue in rats (23). This can be explained by the food, fluid and caloric intake of animals. It has been demonstrated that rats given a 20% fructose solution consume much less water than those given a 10% solution (24). This might explain they are not gaining weight because they are eating fewer calories. There is additional evidence that fructose administration in rats results in a decrease in food consumption (24). Despite fructose consumption, these animals did not gain weight, which might be related to a reduction in food intake because of increased calorie intake.

We tested total cholesterol, HDL, LDL, and triglyceride levels for 16 weeks to see if there were any changes in lipid profile due to high fructose consumption, and only the triglyceride level increased statistically significantly in the HF group. The findings of previous research that showed an increase in triglyceride levels because of fructose consumption are consistent with our findings. This rise might be attributed to a rise in liver triglyceride production as a result of fructose ingestion (25). In addition, the increased plasma triglyceride level can be attributed to decreased adipose tissue triglyceride uptake. Indeed, it has been demonstrated that defective triacylglycerol absorption by adipose tissue, presumably as a result of decreased adipose tissue lipoprotein lipase activity due to decreased insulin excursions, may also contribute to fructose-induced hypertriacylglycerolaemia in humans (26).

The findings of research investigating the impact of excessive fructose intake on lipid profiles are contradictory. Some studies reported that fructose consumption at various times and doses affected the lipid profile (27, 28), while others reported that fructose did not change the lipid profile (29, 30). We observed that increased fructose consumption had no effect on plasma lipid parameters such as total cholesterol, HDL, and LDL, except triglycerides. Our findings support prior research demonstrating that fructose intake does not affect the lipid profile. This might be related to the time period during which fructose is consumed. It was demonstrated in research that 28 days of fructose consumption affected the overall lipid profile and that this impairment was reversible after the 28th day (31).

We discovered that the insulin and GLP-1 plasma levels in the HF group were similar to those in the control group in this research. Excess fructose consumption did not affect baseline GLP-1 and insulin plasma levels. The absence of a difference in insulin levels across experimental groups suggests that consuming 20% fructose has no detrimental effect on glucose metabolism. Interestingly, in the control group, a substantial negative correlation was seen between baseline plasma insulin and GLP-1 levels. This result was also obtained in a study that studied the connection between GLP-1 production and insulin sensitivity in the aftermath of bariatric surgery (32). The negative correlation between plasma insulin and GLP-1 levels seen in the control group may be due to overall insulin sensitivity rather than the incretin effect of GLP-1 in healthy persons. Indeed, the effect of GLP-1 on insulin secretion is known to be glucose-dependent and has been demonstrated mostly in hyperglycemic diabetics. This correlation was seen to be absent in the HF group. This finding may be explained by the fact that excessive fructose consumption results in peripheral insulin resistance, for which GLP-1 is produced at a higher rate than in the control group. Plasma insulin and GLP-1 levels following an oral glucose tolerance test may provide further information about the effect of the HF diet on these parameters. We think that more detailed studies are needed to elucidate the details of this issue.

We found that the most obvious effect of the HF diet was on the hippocampus. This effect was especially on INSR. We observed decreased INSR mRNA expression in the hippocampus of the HF group compared to the C group, but no change in GLP-1R mRNA expression. Numerous studies have established the neuroprotective properties of insulin (and INSR) and GLP-1 (and GLP-1R) (19, 33). According to our results, a reduction in INSR mRNA expression levels may result in hippocampal injury. This may have a detrimental effect on hippocampus neuronal survival. As a result, the hippocampus volume may diminish, and synaptic plasticity may be disrupted in these rats. Consistent with this, a study of rats on a high-calorie fructose diet demonstrated a decrease in the number of neurons in the temporal cortex and hippocampus (34). Taking all of this into account, together with our findings, it is reasonable to conclude that excessive fructose consumption may have a detrimental effect on neurons. For instance, given that increased fructose consumption reduces INSR, brain insulin resistance is most likely to occur. Consistent with our findings, Djordjevic *et al*. demonstrated that chronic ingestion of 10% fructose solution induces hippocampus insulin resistance and inflammation (35). This finding that fructose consumption causes insulin resistance has been confirmed by many researchers. In all of these studies, there was a decrease in the phosphorylation of the insulin receptor (36), insulin receptor substrate 1 (IRS-1) (37), and serine/threonine protein kinase B (Akt) activity (38), and an increase in the phosphorylation of protein tyrosine phosphatase 1B (39), indicating that the insulin signal is impaired due to post-receptor effects, which have been suggested to contribute to neural insulin resistance by dephosphorylating upstream insulin cascade components. To our knowledge, this is the first study to show, at least at the mRNA level, that the cause of insulin resistance produced by fructose consumption may be directly linked to a reduction in the number of insulin receptors, in addition to the previously mentioned altered insulin signaling mechanisms.

When we examined whether the considerable drop in INSR mRNA was mirrored in the protein level, we discovered that the INSR protein level in the hippocampus tended to decrease, but this decline was not statistically significant. It is well known that the amounts of mRNA and protein are not always correlated. Indeed, the association between protein and mRNA expression levels has been shown to be quite low in mammals, with a Pearson correlation value of 0.40 (40). Post-transcriptional regulation, differences in protein half-lives, and measurement noise have been suggested as possible reasons for this weak correlation (41). Due to the limited correlation between protein and mRNA data, integration is difficult. 

In rats consuming fructose, we also observed a decrease in hippocampus GLP-1R mRNA expression, although not statistically significant. Perry *et al*. demonstrated that activating the GLP-1R in the hippocampus formation protects neurons from excitotoxic or beta-amyloid-induced damage (42, 43). GLP-1R is expressed in neural stem/progenitor cells (44) and stimulates hippocampus neurogenesis (45). Reduced expression of these receptors can have a detrimental effect on neuronal survival and neurogenesis in the hippocampus. In rats fed a high fructose diet, hippocampal plasticity was hindered (35). This has been observed to disrupt hippocampus synaptic plasticity and cognitive function (46). Our study’s finding of reduced GLP-1R mRNA expression as a result of excessive fructose consumption corroborated these findings. 

## Conclusion

In summary, 16 weeks of consumption of 20% fructose water had no effect on body weight, fasting plasma GLP-1 and insulin levels, insulin resistance, HDL, LDL, and total cholesterol in Wistar rats.

Additionally, our findings indicated a strong negative correlation between basal plasma insulin and GLP-1 levels in the C group. Fructose ingestion was found to diminish this strong negative correlation. Additionally, in the HF group, fructose intake increased abdominal fat and plasma triglyceride levels. In our study, we found that fructose consumption caused a statistically significant decrement in INRS mRNA level and a tendency to decrease in GLP-1R mRNA level in the hippocampus. 

Additionally, we observed no difference in the protein expression of INSR and GLP-1R in the HF group compared to the C group in the hippocampus. These observations were hypothesized to be involved in mediating the negative neuronal effects of high fructose consumption.

## Authors’ Contributions

S C and V K conceived the study and designed the experiments; F A, M T A, and N E A performed experiments and collected data; F A, S C, E K T, O T, and V K analyzed and interpreted results; F A, S C, and V K prepared the draft manuscript; S C, E K T, O T, and V K supervised and managed the study; all authors reviewed the results and approved the final version to be published. 

## Conflicts of Interest

The authors declare that they have no conflicts of interest.
